# Supplementary material for: Lumpy Skin Disease Virus Genome Sequence Analysis: Putative Spatio-Temporal Epidemiology, Single Gene versus Whole Genome Phylogeny and Genomic Evolution
Source: Viruses. 2023 Jun 28;15(7):1471. doi: 10.3390/v15071471 (PMC10385495; doi:10.3390/v15071471)
Supplement: Supplementary file 1 [file viruses-15-01471-s001.zip › viruses-2466775-Figure S1¿CS5.pptx]

## Slide 1
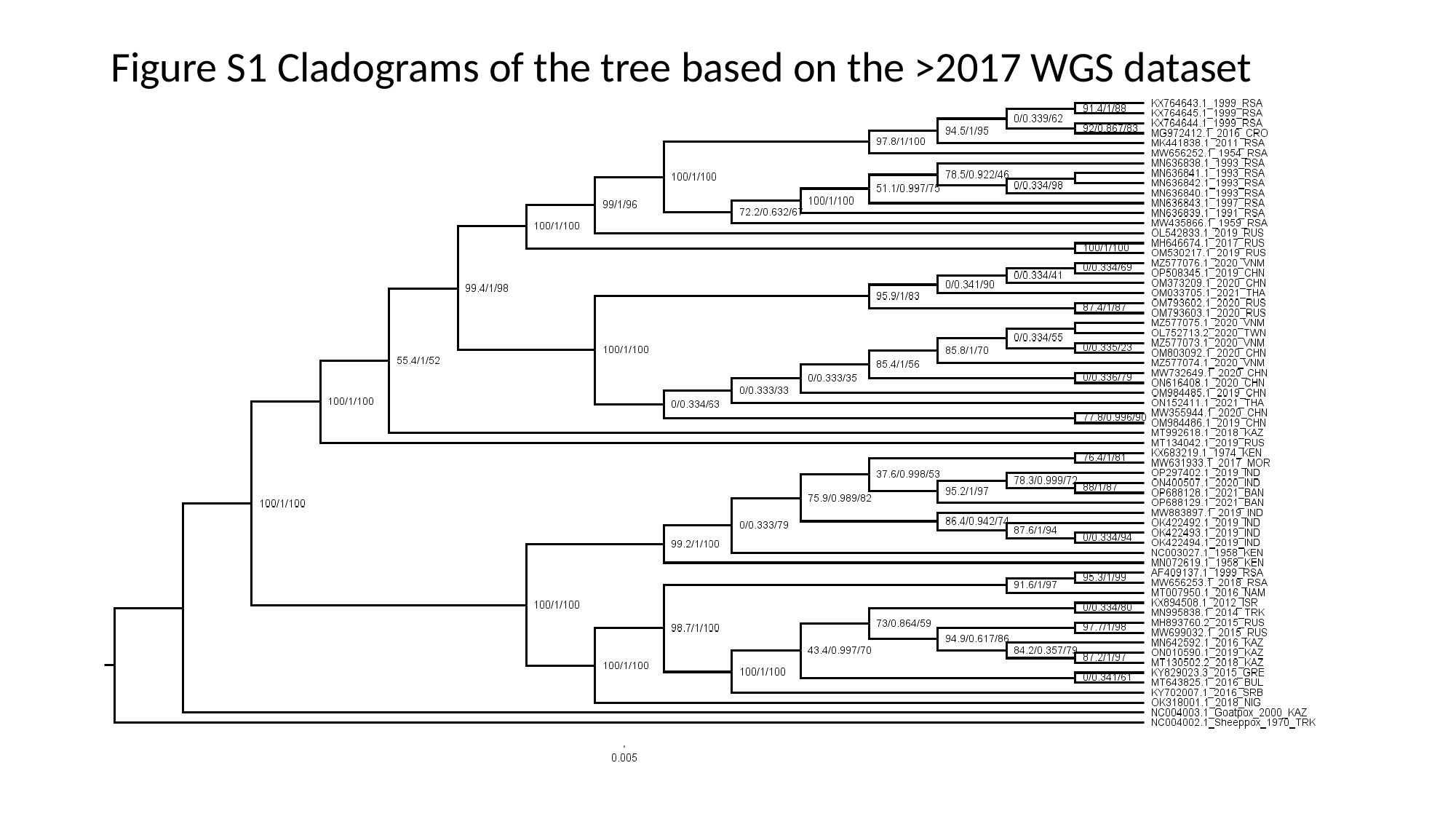

# Figure S1 Cladograms of the tree based on the >2017 WGS dataset

## Slide 2
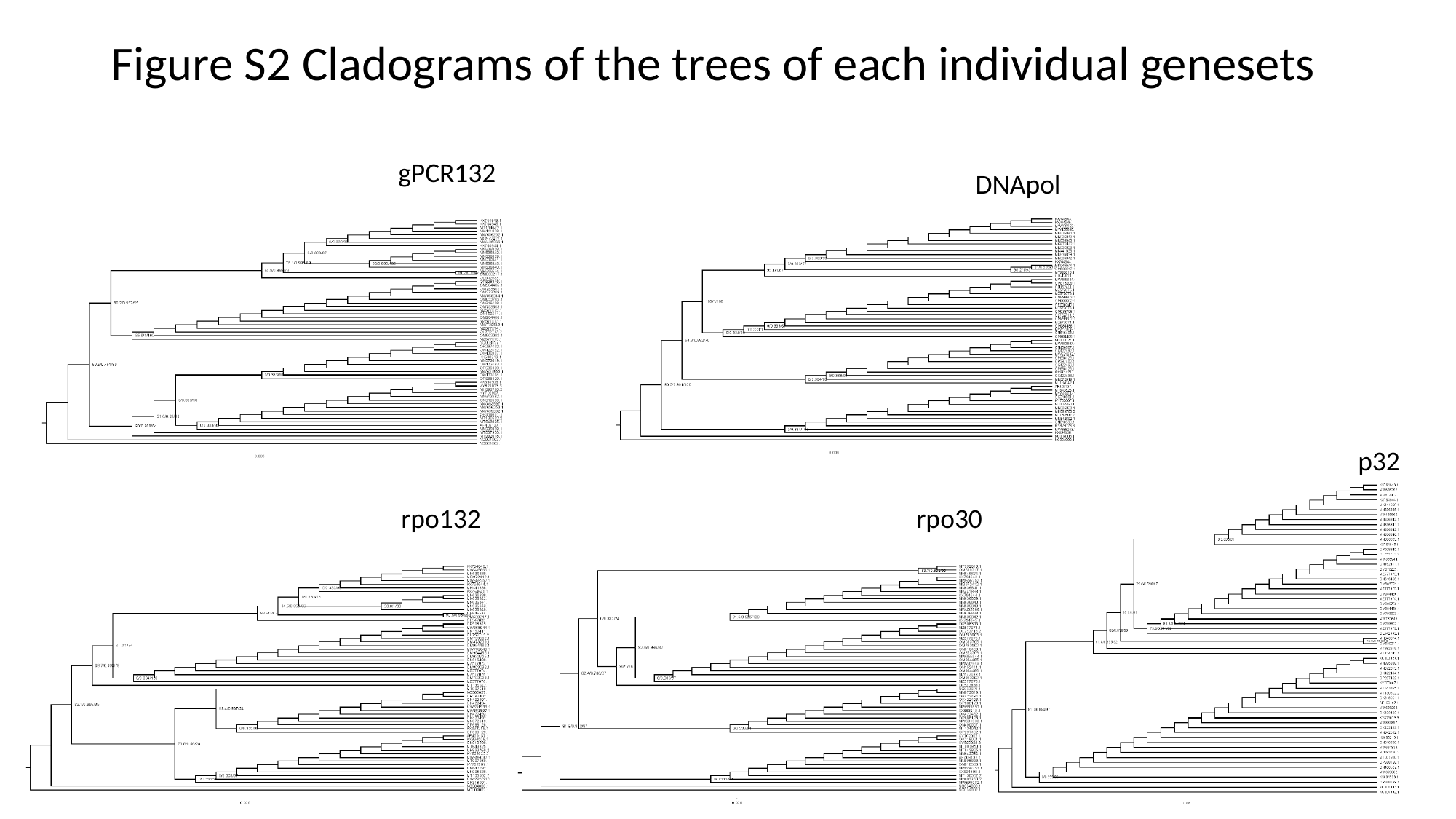

# Figure S2 Cladograms of the trees of each individual genesets
gPCR132
DNApol
p32
rpo132
rpo30

## Slide 3
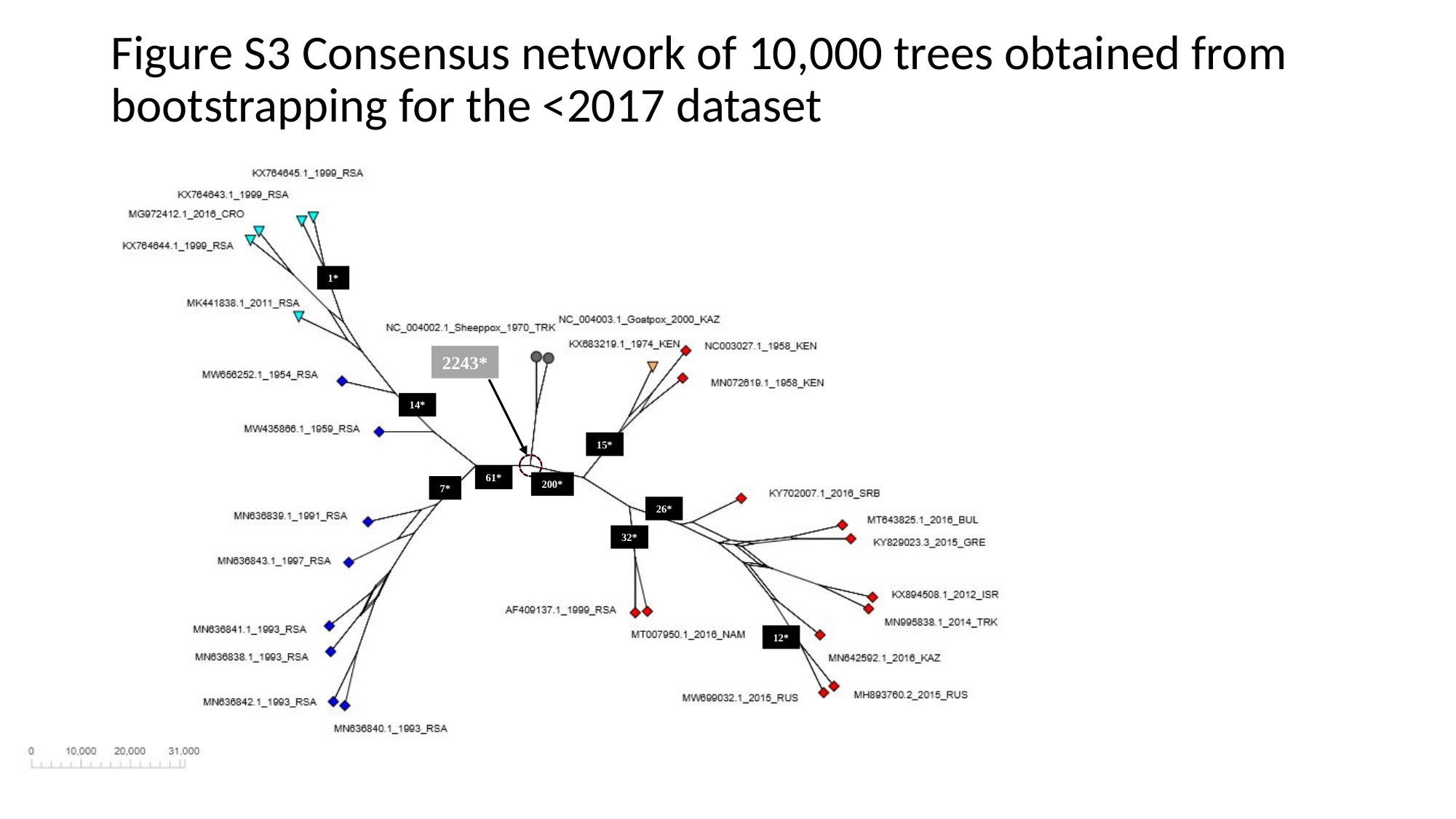

Figure S3 Consensus network of 10,000 trees obtained from bootstrapping for the <2017 dataset
1*
2243*
14*
15*
61*
200*
7*
26*
32*
12*

## Slide 4
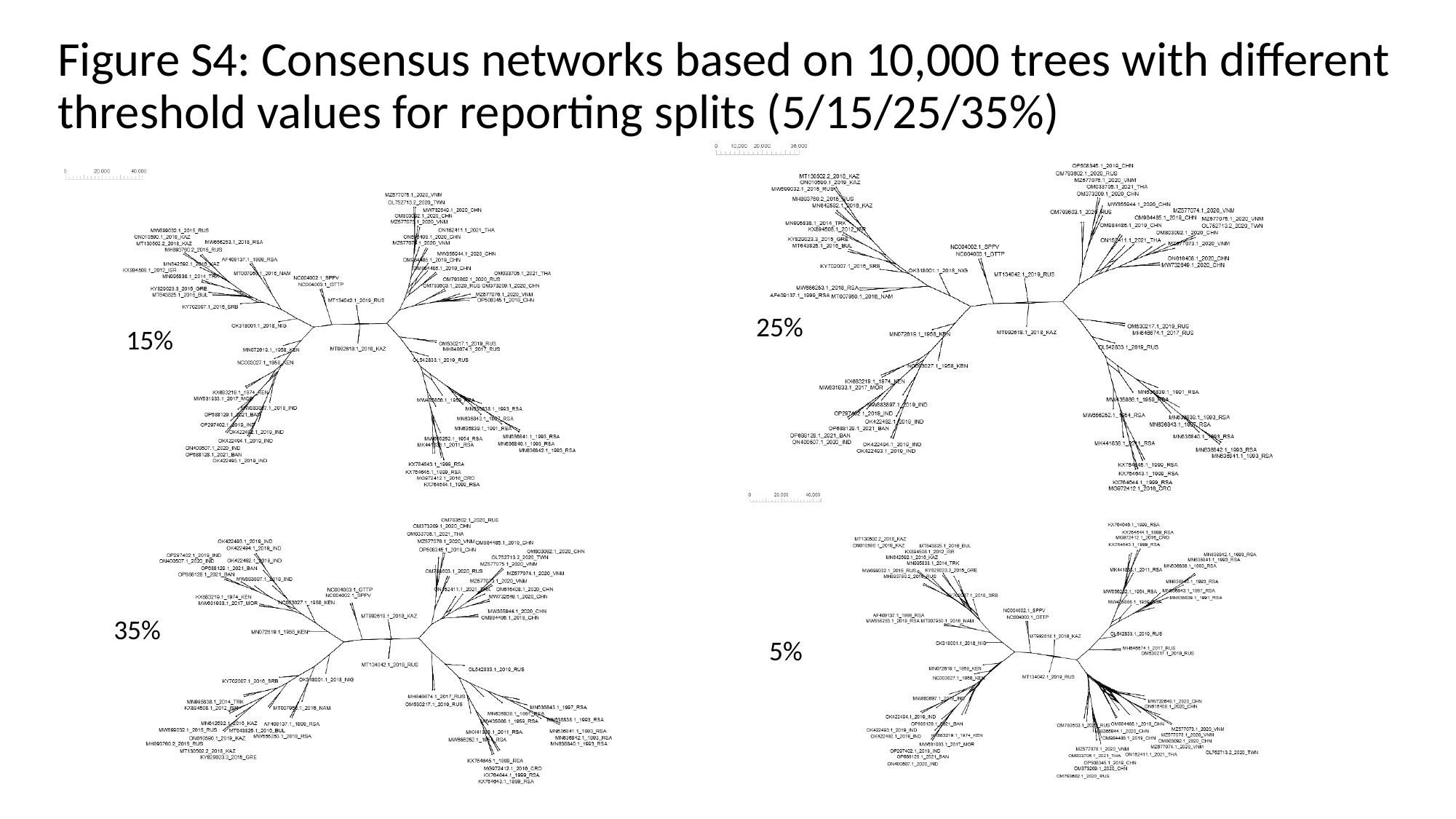

# Figure S4: Consensus networks based on 10,000 trees with different threshold values for reporting splits (5/15/25/35%)
25%
15%
35%
5%

## Slide 5
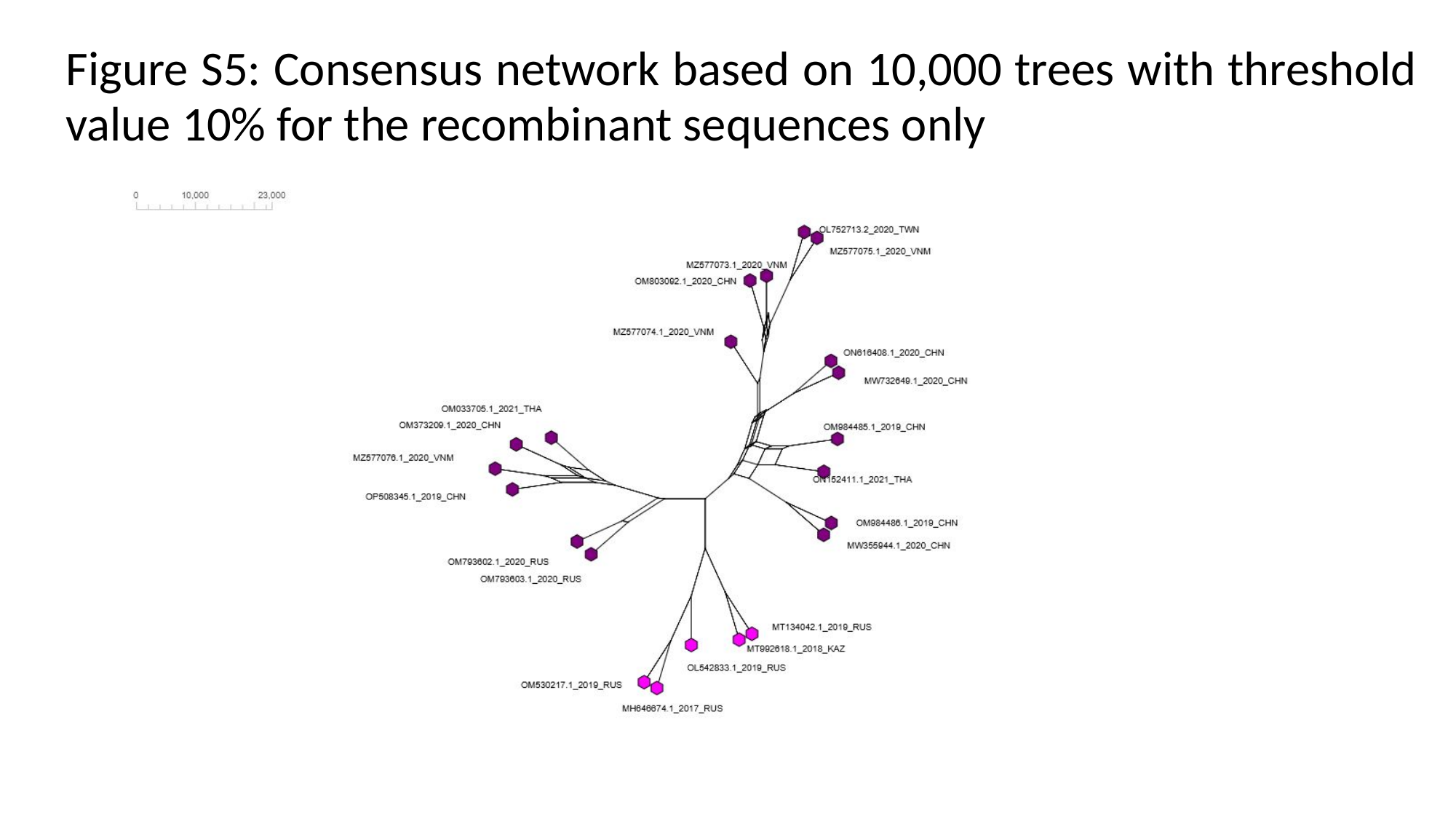

Figure S5: Consensus network based on 10,000 trees with threshold value 10% for the recombinant sequences only
